# Supplementary material for: Novel Driver Strength Index highlights important cancer genes in TCGA PanCanAtlas patients
Source: PeerJ. 2022 Aug 11;10:e13860. doi: 10.7717/peerj.13860 (PMC9375969; doi:10.7717/peerj.13860)
Supplement: Supplemental Information 2 [file peerj-10-13860-s002.zip › COHORTS/genes plots/age/2021_8_16_14_9_distribution_cohorts_75-79.pdf]

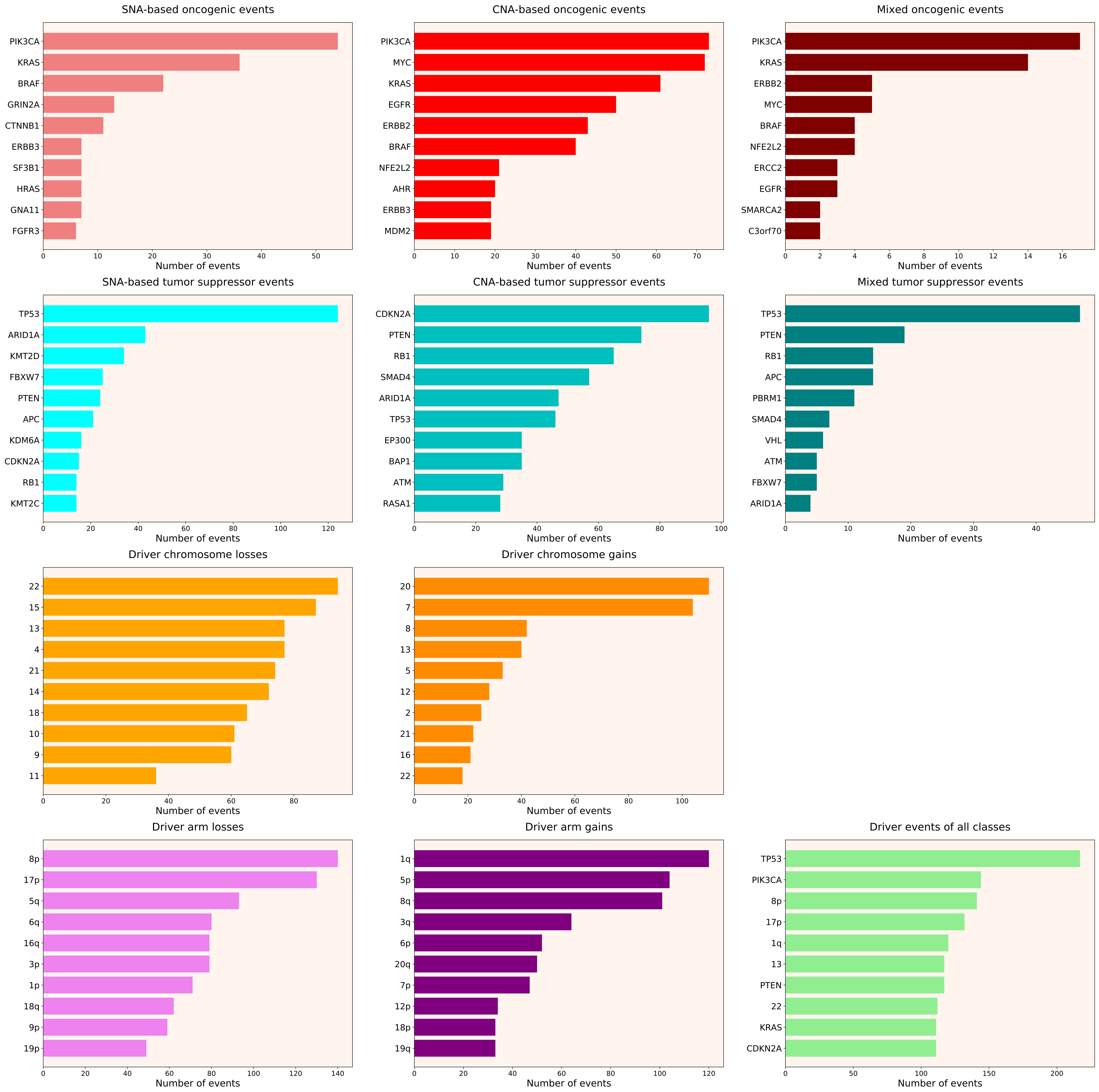
SNA-based oncogenic events
PIK3CA
KRAS
BRAF
GRIN2A
CTNNB1
ERBB3
SF3B1
HRAS
GNA11
FGFR3
0 10 20 30 40 50
Number of events
CNA-based oncogenic events
PIK3CA
MYC
KRAS
EGFR
ERBB2
BRAF
NFE2L2
AHR
ERBB3
MDM2
0 10 20 30 40 50 60 70
Number of events
Mixed oncogenic events
PIK3CA
KRAS
ERBB2
MYC
BRAF
NFE2L2
ERCC2
EGFR
SMARCA2
C3orf70
0 2 4 6 8 10 12 14 16
Number of events
SNA-based tumor suppressor events
TP53
ARID1A
KMT2D
FBXW7
PTEN
APC
KDM6A
CDKN2A
RB1
KMT2C
0 20 40 60 80 100 120
Number of events
CNA-based tumor suppressor events
CDKN2A
PTEN
RB1
SMAD4
ARID1A
TP53
EP300
BAP1
ATM
RASA1
0 20 40 60 80 100
Number of events
Mixed tumor suppressor events
TP53
PTEN
RB1
APC
PBRM1
SMAD4
VHL
ATM
FBXW7
ARID1A
0 10 20 30 40
Number of events
Driver chromosome losses
22
15
13
4
21
14
18
10
9
11
0 20 40 60 80
Number of events
Driver chromosome gains
20
7
8
13
5
12
2
21
16
22
0 20 40 60 80 100
Number of events
Driver arm losses
8p
17p
5q
6q
16q
3p
1p
18q
9p
19p
0 20 40 60 80 100 120 140
Number of events
Driver arm gains
1q
5p
8q
3q
6p
20q
7p
12p
18p
19q
0 20 40 60 80 100 120
Number of events
Driver events of all classes
TP53
PIK3CA
8p
17p
1q
13
PTEN
22
KRAS
CDKN2A
0 50 100 150 200
Number of events
